# Multicenter Prospective Cohort Study of the Diagnostic Yield and Patient Experience of Multiplex Gene Panel Testing For Hereditary Cancer Risk

**DOI:** 10.1200/PO.18.00217

**Published:** 2019-03-28

**Authors:** Gregory E. Idos, Allison W. Kurian, Charité Ricker, Duveen Sturgeon, Julie O. Culver, Kerry E. Kingham, Rachel Koff, Nicolette M. Chun, Courtney Rowe-Teeter, Alexandra P. Lebensohn, Peter Levonian, Katrina Lowstuter, Katlyn Partynski, Christine Hong, Meredith A. Mills, Iva Petrovchich, Cindy S. Ma, Anne-Renee Hartman, Brian Allen, Richard J. Wenstrup, Johnathan M. Lancaster, Krystal Brown, John Kidd, Brent Evans, Bhramar Mukherjee, Kevin J. McDonnell, Uri Ladabaum, James M. Ford, Stephen B. Gruber

**Affiliations:** ^1^ University of Southern California, Los Angeles, CA; ^2^ Stanford University School of Medicine, Stanford, CA; ^3^ Myriad Genetics, Salt Lake City, UT; ^4^ University of Michigan, Ann Arbor, MI

## Abstract

**Purpose:**

Multiplex gene panel testing (MGPT) allows for the simultaneous analysis of germline cancer susceptibility genes. This study describes the diagnostic yield and patient experiences of MGPT in diverse populations.

**Patients and Methods:**

This multicenter, prospective cohort study enrolled participants from three cancer genetics clinics—University of Southern California Norris Comprehensive Cancer Center, Los Angeles County and University of Southern California Medical Center, and Stanford Cancer Institute—who met testing guidelines or had a 2.5% or greater probability of a pathogenic variant (N = 2,000). All patients underwent 25- or 28-gene MGPT and results were compared with differential genetic diagnoses generated by pretest expert clinical assessment. Post-test surveys on distress, uncertainty, and positive experiences were administered at 3 months (69% response rate) and 1 year (57% response rate).

**Results:**

Of 2,000 participants, 81% were female, 41% were Hispanic, 26% were Spanish speaking only, and 30% completed high school or less education. A total of 242 participants (12%) carried one or more pathogenic variant (positive), 689 (34%) carried one or more variant of uncertain significance (VUS), and 1,069 (53%) carried no pathogenic variants or VUS (negative). More than one third of pathogenic variants (34%) were not included in the differential diagnosis. After testing, few patients (4%) had prophylactic surgery, most (92%) never regretted testing, and most (80%) wanted to know all results, even those of uncertain significance. Positive patients were twice as likely as negative/VUS patients (83% *v* 41%; *P* < .001) to encourage their relatives to be tested.

**Conclusion:**

In a racially/ethnically and socioeconomically diverse cohort, MGPT increased diagnostic yield. More than one third of identified pathogenic variants were not clinically anticipated. Patient regret and prophylactic surgery use were low, and patients appropriately encouraged relatives to be tested for clinically relevant results.

## INTRODUCTION

Genetic testing is a powerful tool to stratify cancer risk. Recent advances in massively parallel sequencing have expanded germline testing for hereditary cancer risk assessment.^1-3^ Multiplex gene panel testing (MGPT) simultaneously analyzes multiple genes and may provide an advantage over sequential single-gene testing in terms of cost, speed, and clinical utility. As a result of the wide spectrum and considerable overlap of phenotypes associated with hereditary cancer syndromes, MGPT offers a potentially practical and efficient approach with which to identify the genetic cause of inherited cancer susceptibility.^4-13^

Despite these advantages, MGPT has raised concerns about the identification of pathogenic variants that do not correlate with phenotype—for example, *BRCA1* or *BRCA2* mutations in patients clinically suspected to have Lynch syndrome.^14^ Furthermore, MGPT yields 10-fold higher rates of clinically ambiguous variants of uncertain significance (VUS) compared with more limited testing approaches.^2-13,15,16^ Little is known about the effect of this greater volume of uncertain results on the outcomes and patient experience of cancer risk assessment, and it is crucial to learn more as more comprehensive sequencing approaches on the horizon.

**CONTEXT**

What are the benefits, harms, and patient experiences after germline multiplex gene panel testing for cancer susceptibility?
Among 2,000 participants in this fully accrued, multicenter, prospective cohort study of hereditary cancer testing with a 25- or 28-gene panel, more than one quarter of the pathogenic variants identified were not clinically anticipated. Multiplex genetic panel testing for hereditary cancer risk assessment substantially increases the diagnostic yield of germline mutations compared with expert differential diagnosis. Reassuringly, at a median follow-up of 13 months, patients did not regret having undergone multiplex testing, did not overuse preventive surgery, and encouraged their family members to be tested in accordance with practice guidelines.
Multiplex genetic panel testing enhances the diagnostic yield of genetic testing without discernible harm to patients. This study also demonstrates that testing at lower predicted levels of pathogenic variant carriage—a probability threshold of 2.5%—which reflects recent changes in clinical practice and guidelines, is effective and safe. Overall, this study offers significant and novel results on the performance of multiplex genetic testing and has broad implications for its clinical implementation and acceptance.

We designed a prospective cohort study of hereditary cancer testing with a multigene panel to measure the benefits, harms, and patient experiences of MGPT. Our hypotheses were that MGPT with pretest genetic counseling would be associated with patient regret, use of preventive surgery, and family communication of results. We report the diagnostic yield and patient experience of MGPT after full accrual of the planned 2,000 participants.

## PATIENTS AND METHODS

### Study Population

The study was approved by institutional review boards (protocol #HS-13-00431) at the University of Southern California (USC) and Stanford University. Written informed consent was obtained in person from each patient. Details on methods are reported in the Data Supplement. Participants were consecutively recruited between July 2014 and November 2016 at three medical centers—USC Norris Comprehensive Cancer Center, Los Angeles County and USC Medical Center, and Stanford University Cancer Institute. Eligible patients met clinical guideline criteria for genetic testing or had a 2.5% or greater probability of carrying mutation calculated by the following validated models or algorithms: BOADICEA, BRCAPro, IBIS (Tyrer-Cuzick), PANCPro, PREMM_1,2,6_, PENN II, PTEN Cleveland Clinic Score, MELAPro, MMRPro, Myriad II, National Comprehensive Cancer Network guidelines, or a personal history of more than 10 cumulative lifetime colorectal adenomas.

### Next-Generation Sequencing Assay

MGPT was accomplished with a multiple-gene, next-generation sequencing (NGS) test performed by Myriad Genetic Laboratories (Salt Lake City, UT), which included testing for *APC, ATM, BARD1, BMPR1A, BRCA1, BRCA2, BRIP1, CDH1, CDK4, CDKN2A, CHEK2, EPCAM, GREM1, MLH1, MSH2, MSH6, MUTYH, NBN, PALB2, PMS2, POLD1, POLE, PTEN, RAD51C, RAD51D, SMAD4, STK11,* and *TP53* (Data Supplement). All genes were included over the full study period with the exception of *GREM1*, *POLD1*, and *POLE*, which were added in July 2016. There were 1,664 participants who underwent 25-gene panel testing and 336 who underwent 28-gene panel testing. Sequencing and large rearrangement analysis were performed for all genes, except *POLD1* and *POLE* (sequencing only) and *EPCAM* and *GREM1* (large rearrangement only).

Sample preparation for NGS was performed from frozen DNA using the RainDance microdroplet polymerase chain reaction (PCR) system (RainDance Technologies, Billerica, MA).^17^ In brief, PCR products that represented exons and proximal splicing elements of patient DNA were amplified in merged droplets that consisted of fragmented patient DNA and select target enrichment primers. These PCR products were subsequently tagged with barcodes and sequencing adaptors for NGS on the Illumina HiSeq platform (Illumina, San Diego, CA). To circumvent highly homologous pseudogenes, we used modified sample preparation with long-range and nested PCR, followed by NGS on the Illumina MiSeq platform, for portions of *CHEK2* and *PMS2*. All clinically actionable variants identified by NGS, as well as regions that did not meet preset NGS quality metrics, were independently confirmed with orthogonal site-specific Sanger sequencing. To detect exonic deletions and duplications, NGS dosage, microarray comparative genomic hybridization, multiplex ligation-dependent probe amplification, or a combination of these analyses was performed, with all positive results confirmed by an orthogonal method.^17^

### Variant Classification

Variants were classified using American College of Medical Genetics and Genomics recommendations, with supporting linkage, biochemical, clinical, functional, and statistical data used for specific missense and intronic alterations.^18-20^ Gene variants classified as deleterious or that were suspected to be deleterious were considered pathogenic. Variants with unknown clinical significance were considered VUS. Variants classified as polymorphism or that favored polymorphism were considered benign.

### Differential Diagnoses

Differential diagnoses were generated for each participant after expert clinical genetics assessment, in which up to eight inherited cancer syndromes were ranked by estimated likelihood using such factors as personal and family cancer history, tumor characteristics, and physical examination^21^ (Data Supplement). The genetics clinician then clarified a level of suspicion for each syndrome in the differential diagnosis by stating whether she or he would test for that syndrome specifically if MGPT were not available.

### Questionnaire Procedures

Participants were invited to answer a baseline questionnaire at the time of their genetics evaluation, with follow-up questionnaires 3, 6 and 12 months after disclosure of MGPT results. Participants were contacted by mail and/or e-mail to complete follow-up questionnaires, which included a brief reminder of the study’s purpose and procedures. All mailed questionnaires included a postage-paid, preaddressed envelope. E-mail participants who did not respond to the initial invitation link received two e-mail reminders over a course of 2 weeks before receiving a reminder phone call from study personnel. Mail participants were given 2 weeks to respond, after which they received a reminder phone call. Using a standardized script, all participants who received a reminder phone call were given the option to receive another e-mail invitation, another mailed paper questionnaire, or to complete the questionnaire over the phone. A maximum of three phone attempts for contacting the participant, including leaving voicemails, were made by study personnel. Participants were considered nonresponders if they had not completed their questionnaire 2 months after the initial send date.

### Patient-Reported Experiences

We used the validated Multidimensional Impact of Cancer Risk Assessment (MICRA)^22^ instrument, which contains subscales that measure distress, uncertainty, and positive experiences in relation to genetic testing. The distress subscale—six items, score range of 0 to 30—evaluates adverse psychological feelings of anxiety and regret. The uncertainty subscale—nine items, score range of 0 to 45—evaluates doubt and frustration. The positive experience subscale—four items, score range of 0 to 20—evaluates relief and satisfaction. Additional questions evaluated intrusive thoughts about cancer and regret about having undergone MGPT, participants’ desired amount of MGPT results information, participants’ notification of relatives about MGPT results, and relatives’ genetic testing behaviors. Participants were also asked if they had undergone specific surgical procedures (mastectomy, salpingo-oophorectomy, or hysterectomy) and the reason for these procedures (cancer treatment, cancer prevention, or other).^23^

### Statistical Analysis

The primary aim of this study was to test the impact of genetic test results—positive, VUS, or negative—on patient experience. MICRA subscale scores were used for this aim. Patients with one or more pathogenic variant were considered positive, whereas those with only benign or uncertain variants were considered VUS and those with only benign variants were considered negative. Sample size estimation was based on comparisons between genetic test results. Assuming a pathogenic variant prevalence of 10%, a VUS prevalence of 35%, and a negative test prevalence of 55%, with the standard deviation of MICRA scores being 4, a sample size of 2,000 patients was needed to achieve more than 80% power to detect a difference of 1 in MICRA scores.

Data analysis was based on information gathered as of March 19, 2018. Descriptive statistics were calculated for demographics, differential diagnoses, surgery, and MGPT results. We used the Pearson χ^2^ test to assess the association between genetic test results and survey responses. Negative binomial regression with a log link was used to analyze the association of genetic test results with MICRA subscale scores while adjusting for covariates that included clinical site, age, gender, ethnicity, education level, and personal history of cancer. *P* values less than .05 were considered statistically significant. All statistical analyses were performed with SAS software (SAS/STAT User’s Guide, Version 9.4; SAS Institute, Cary, NC) and R software (version 3.2.2).

## RESULTS

### Study Population

Two thousand participants were enrolled between July 2014 and November 2016 (Data Supplement). The majority of patients were female (81%) and 73% had a personal history of cancer (Table 1). Common reasons for genetics referral include the following: cancer diagnosis at age 50 years or younger (39%), two or more first- or second-degree relatives with cancer (64%), and one or more family members diagnosed with cancer at younger than age 50 years (63%). Participants were diverse both racially/ethnically and sociodemographically—39% were Hispanic (primarily of Mexican or Central American ancestry), 40% non-Hispanic white,12% Asian (primarily of Chinese and Filipino ancestry), and 4% black. Approximately one quarter (26%) spoke Spanish as their primary language, and 30% had completed high school or less education (Table 1).

**TABLE 1.**
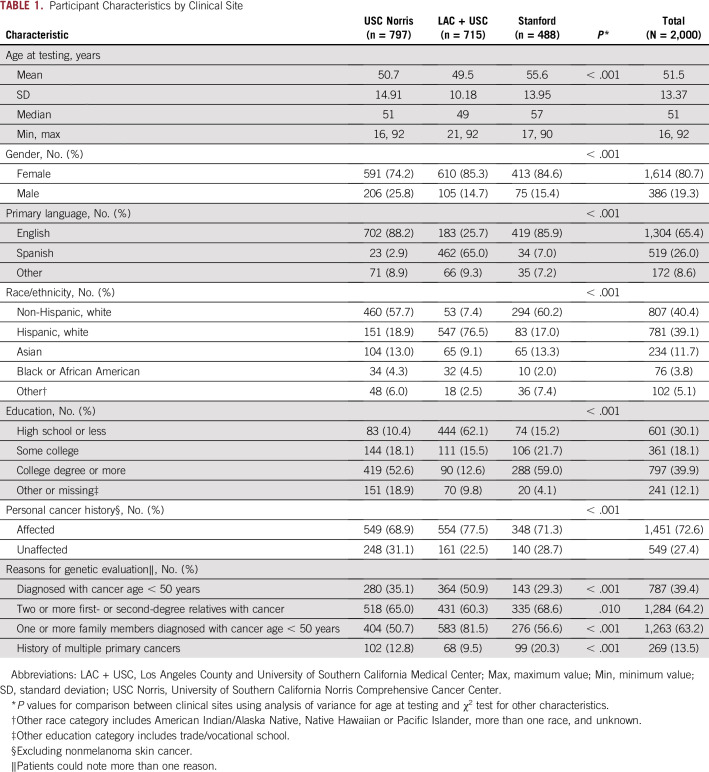
Participant Characteristics by Clinical Site

### Frequency of Pathogenic Variants and VUS

**FIG 1.**
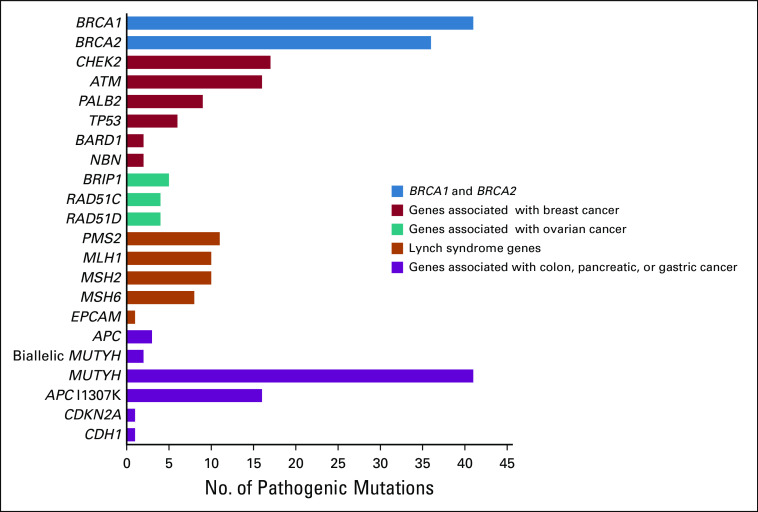
Overall yield of genetic testing among 2,000 participants. Cancer associations listed here are not comprehensive and include the highest risk or primary clinical indication.

At least one pathogenic variant was identified in 242 participants (12%; Fig 1). Seventy-six patients—31% of all pathogenic variant carriers—had a germline mutation in *BRCA1* and/or *BRCA2*, and 39 (16%) had a pathogenic variant in a mismatch repair gene conferring a diagnosis of Lynch syndrome. Forty-three patients (18%) had a pathogenic *MUTYH* variant, monoallelic (n = 41) or biallelic (n = 2). Nineteen patients (8%) had pathogenic variants in *APC*, with 16 of them having the founder mutation *APC* I1307K. Six patients had pathogenic variants in *TP53* (2%). Other genes in which pathogenic variants were detected included *CHEK2* (n = 17; 7%), *ATM* (n = 16; 7%), *PALB2* (n = 9; 4%), *BRIP1* (n = 5; 2%), *RAD51C* (n = 4; 2%), *BARD1* (n = 2; 1%), *NBN* (n = 2; 1%), *CDH1* (n = 1; 0.4%), and *CDKN2A* (n = 1; 0.4%). Pathogenic variant status and associated patient characteristics are listed in Table 2. Among patients without pathogenic variants, 689 (34%) had at least one VUS, with up to four per patient (Table 2).

**TABLE 2.**
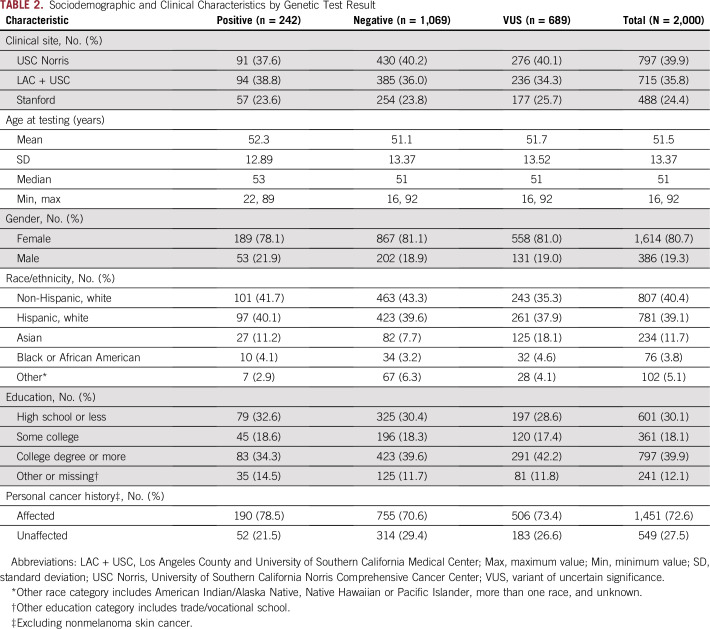
Sociodemographic and Clinical Characteristics by Genetic Test Result

### Differential Diagnosis Versus MGPT Results

Of 242 participants with pathogenic variants, 160 (66%) had pathogenic variants in genes related to syndromes that were included in the pretest differential diagnosis. Eighty-two patients (34%) had pathogenic variants that were not clinically anticipated—monoallelic *MUTYH* (n = 32; 39%), *APC I1307K* (n = 13; 16%), *CHEK2* (n = 10; 12%), *PALB2* (n = 8; 10%), *ATM* (n = 7; 9%), *BRIP1* (n = 4; 5%), *BRCA2* (n = 3; 4%), *BRCA1* (n = 2; 2%), *PMS2* (n = 2; 2%), and *TP53* (n = 1; 1%).

### Use of Surgery After MGPT

At a median follow-up of 13 months, 198 (13%) of 1,573 returning surveys reported undergoing surgery after MGPT. Surgery rates and stated reasons were as follows: mastectomy (n = 162 [11.3%]: 90% for cancer treatment, 30% for cancer prevention, and 2% for benign breast disease), salpingo-oophorectomy (n = 43 [3%]: 27% for cancer treatment and 56% for cancer prevention), and hysterectomy (n = 23 [2%]: 50% for cancer treatment, 18% for cancer prevention, and 9% for benign disease). Overall, only 4% (n = 62) of patients underwent a prophylactic operation. Significantly more patients who tested positive (n = 30; 16%) had preventive surgery compared with either patients testing negative (n = 20; 2.4%; *P* < .001) or those with a VUS (n = 12; 2.3%; *P* < .001). There was no significant difference in the use of preventive surgery between those testing negative compared with VUS (*P* = .919). To illustrate, there were 10 patients identified as having high-penetrance founder mutations in *BRCA1* or *BRCA2*, six of whom were found to have undergone or were considering risk-reducing mastectomy or oophorectomy. In comparison, there were 16 patients identified as carriers of the low-penetrance *APC* I1307K allele, none of whom had undergone an inappropriate surgery (Data Supplement).

### Patient Experiences With MGPT

At a median follow-up of 4 months, the response rate was 69%. Overall, levels of genetic testing–specific concerns were low. Mean scores for each MICRA subscale—distress, uncertainty, or positive experiences—all differed significantly between patients with positive test results compared with the group of patients with a negative or VUS result (Table 3). Compared with patients testing negative or VUS, patients who tested positive had significantly higher distress scores (*P* < .001), significantly higher uncertainty scores (*P* < .001), and significantly lower positive experiences scores (*P* < .001 or *P* = .007, respectively). Compared with patients testing negative, patients with a VUS had significantly higher uncertainty scores (*P* = .017), but not significantly different distress or positive experiences scores (*P* = .249 or *P* = .399, respectively).

**TABLE 3.**
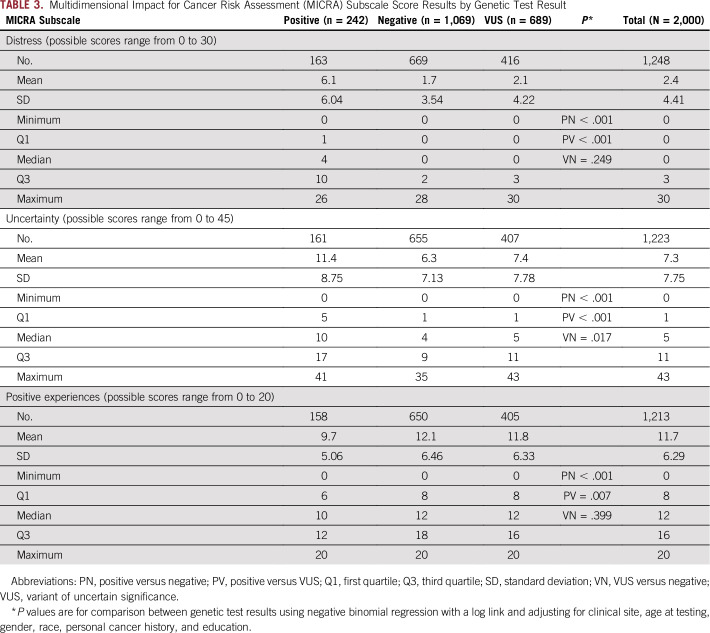
Multidimensional Impact for Cancer Risk Assessment (MICRA) Subscale Score Results by Genetic Test Result

**FIG 2.**
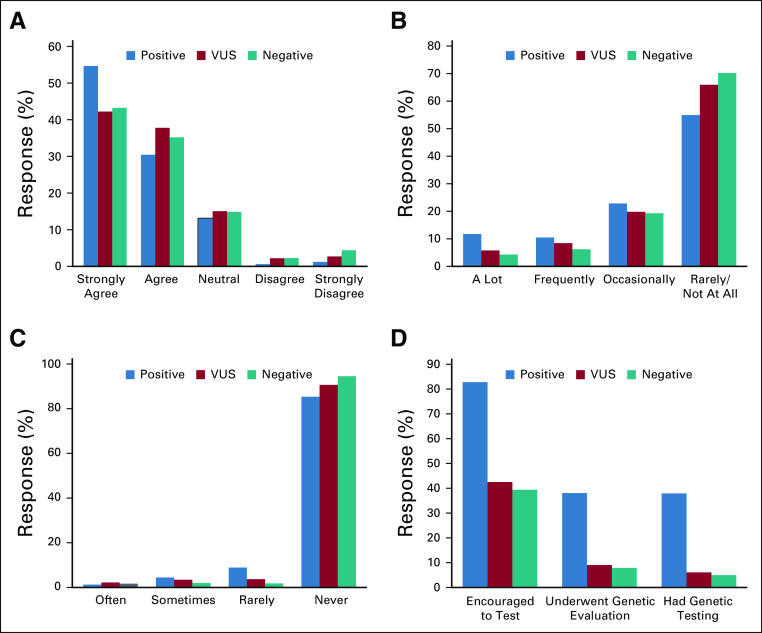
Patient experiences after multiplex testing. (A) I want to know everything, including findings that doctors do not fully understand. (B) Thoughts of cancer affect my daily activities. (C) I regret learning about my test results. (D) Family notification of genetic testing.

Two thirds of patients (67%) stated that thoughts of cancer rarely or never affected their daily activities, most (92%) never regretted learning their MGPT results, and most (80%) wanted to know all MGPT results, including findings that doctors do not fully understand (Fig 2). Responses to these questions did differ significantly between patients with positive and negative results, with the exception of wanting to know all MGPT results. A higher proportion of patients testing negative as compared to positive reported rarely or never having thoughts of cancer affect their daily activities (70% *v* 55%; *P* < .001). Also, a higher proportion of patients testing negative as compared to positive reported that they did not regret learning their test results (95% *v* 85%; *P* < .001; Fig 2). However, all were similarly likely (96% to 99%) to notify relatives about results. There were significant differences (*P* < .001) in family communication and encouragement of relatives’ testing between positive (for whom relatives’ testing is strongly indicated)^21,22^ versus the VUS or negative group (for whom relatives’ testing is sometimes indicated)^21,22^ as follows: encouraged relatives to have genetic testing (positive, 83%; VUS, 43%; negative, 40%), relatives underwent genetic testing (positive, 38%; VUS, 6%; negative, 5%), and relatives underwent screening because of increased cancer risk (positive, 45%; VUS, 23%; negative, 19%).

## DISCUSSION

This prospective cohort study of MGPT for hereditary cancer risk presents data that inform the current practice of genetic testing. Results offer a window into patient perceptions of a future driven by increasingly complex and rich genetic information. Among patients who carried a pathogenic variant (12%), one third of these clinically relevant results were not suspected upon pretest expert assessment. We found little evidence of patient harm and substantial evidence that patients understood the meaning of their MGPT results, as patients who tested positive for a pathogenic variant were more likely to encourage appropriate genetic testing in relatives compared with those who tested with VUS or negative results. These results suggest an important contribution of MGPT to clinical cancer risk assessment of probands and their families.

A striking result was that one in three identified pathogenic variants was missed in the differential diagnoses generated by expert clinicians. This finding highlights the limitations of clinical risk assessment and the incremental diagnostic yield from testing genes beyond those implicated in a single syndrome. Of note, few of the missed variants were in genes associated with well-known syndromes—for example, Lynch syndrome, hereditary breast/ovarian cancer—which likely reflects clinicians’ greater familiarity with their presentation. Instead, most were in moderate-penetrance genes that confer a two- to four-fold increase in cancer risk (eg, *ATM* and *CHEK2)* as well as low-penetrance mutations (eg, monoallelic *MUTYH* and *APC* I1307K). Given the short history of widespread clinical testing of these genes,^24-27^ their phenotype is less well characterized and more difficult for clinicians to recognize. Moreover, our relatively low threshold for study eligibility—estimated risk of pathogenic variant probability of 2.5% of greater, approximately one half that of practice guidelines,^28,29^ chosen using published cost-effectiveness estimates at different risk thresholds^30^—may have enriched for less-penetrant variants. Some questions remain about the clinical benefit of detecting low- to moderate-penetrance pathogenic variants; however, these variants meet criteria for intensified cancer screening according to current practice guidelines,^28,29^ which makes them relevant to patient care. By identifying unexpected pathogenic variants, MGPT can broaden our understanding of genotype-phenotype correlations and the spectrum of associated cancer risks.

Concerns have been raised about high rates of identifying VUS in MGPT. Consistent with prior studies, we report a VUS rate of 34%.^5-8^ Recent studies found limited genomic confidence among oncologists and that few (less than 15%) community physicians who order *BRCA1* and *BRCA2* tests understand the correct management of VUS.^27,31,32^ We recently published that patients who receive VUS results from genetic testing are substantially more likely to seek a second opinion from a new medical oncologist.^33^ A related concern is that MGPT results of unknown clinical relevance, whether VUS or a pathogenic variant whose cancer risk is insufficiently characterized, might prompt invasive and irreversible prophylactic operations.^34-36^ Data from our prospective cohort study do not support this hypothetical concern. Reassuringly, we found that prophylactic operations were not overused—only 4% underwent prophylactic mastectomy, hysterectomy, or salpingo-oophorectomy, and these procedures were no more frequent among patients with VUS results than those with negative results. With a median follow-up time of 13 months after genetic testing, these results do not suggest an overuse of surgery after MGPT in this study.

Of note, overall yield of pathogenic variants in this study is 12%, which is similar to other studies. Whereas panels may vary in the number of genes tested, all large panels, including the one used in this study, include genes that are associated with known syndromes—that is, hereditary breast/ovarian cancer and Lynch syndrome—which account for the majority of pathogenic mutations identified in most multigene panel studies. Our group previously examined the additional yield of mutations identified via multigene panel testing and found that by increasing the number of genes tested, the frequency of mutations identified also increased.^37,38^ Independently, a recent study by Mandelker et al^39^ demonstrated a 17.5% yield of pathogenic variants after sequencing 1,040 patients with advanced cancer using the Memorial Sloan Kettering IMPACT panel of 410 genes.

Patient-reported experiences were also reassuring. Most patients never regretted undergoing MGPT or had intrusive thoughts about cancer, and most wanted to know all MGPT results, even those that physicians do not fully understand. This is concordant with prior studies that have demonstrated that patients consistently express a desire to know genetic test results in a multitude of settings.^40^ We found that MICRA subscale scores varied significantly between patients who tested positive compared with those who had a negative or VUS test result, but scores did not vary between patients with a VUS versus negative except in the category of uncertainty. Patients also communicated differently to relatives. Those who tested positive were twice as likely to urge their relatives to be tested, which is guideline concordant and appropriate management for a positive result. These findings suggest that patients rarely misinterpret VUS with appropriate pretest genetic counseling as most VUSs that are reclassified are ultimately reclassified to benign.^15,41^ On the contrary, these findings suggest that patients understood the implications of their MGPT results, which is particularly encouraging in our population as approximately one third received high school education or less. The present results contrast with our recent finding of gaps in the understanding and management of VUS in community practice, and serve to demonstrate the value of pre- and post-test counseling with proper anticipatory guidance by a trained professional.^42-44^

There are limited published data on pathogenic mutation rates among Hispanics after multiplex gene panel testing, as Hispanics are underrepresented in a majority of clinical studies. Of note, we found that 12.1% (n = 97 of 781) of Hispanic patients had a pathogenic variant. In our previous USC-based paper by Ricker et al,^37^ in which Hispanic patients made up 47.6% (n = 228) of the cohort, there was a pathogenic mutation frequency of 14.1% (n = 33). In a recent publication from Stanford, Caswell-Jin et al^45^ reported that 21% of their 213 Hispanic participants carried a pathogenic variant. In a study by Rosenthal et al,^11^ among the 8% (n = 19,795) of Hispanic patients who underwent multigene panel testing, 6.7% (n = 1,326) were found to have a pathogenic mutation. More data are needed to understand the rates of pathogenic mutations in different ethnic groups.

Aspects of our study merit comment. Its considerable strengths include a prospective, multicenter design; a diverse population in terms of race/ethnicity, language, and education; a high survey response rate; and uniform pretest assessment by experienced genetic counselors. The participation rate of Hispanic/Latino, African American, and Asian patients was high compared with other published studies. To our knowledge, it is the first study to demonstrate that testing at lower predicted levels of pathogenic variant carriage, which reflects recent changes in clinical practice and guidelines, is effective and safe. This study offers significant and novel results on the performance of multiplex genetic testing and has broad implications for its clinical implementation and acceptance. Looking forward, interim analysis of our data suggests that patients who are found to have a pathogenic variant in a high- or moderate-risk gene are more likely to undergo appropriate screening and surveillance compared with those who tested negative or with a VUS within 1 year of MGPT testing. For example, patients who were identified as carrying a pathogenic variant in a gene associated with Lynch syndrome were four times as likely (*P* < .001) to undergo colonoscopy compared with patients with a VUS or negative genetic test result. Encouragingly, this trend is consistent when we stratify by ethnicity in our Hispanic cohort.

Its limitations include 13 months of follow-up time to date. In addition, our survey response rate decreased to 57% at 12 months from 69% at 3 months, as we primarily recruited from a clinical cohort, most of whom were affected with cancer and undergoing active treatment. During the course of follow-up, some patients died, moved to another location, or changed their contact information. Nonetheless, this fully accrued, prospective study of 2,000 participants has immediate relevance for patient care.

In conclusion, the results of this prospective cohort study support the use of multiplex gene panel testing for hereditary cancer risk assessment with appropriate genetic counseling, and demonstrate its capacity to enhance the diagnostic yield of genetic testing without discernible harm to patients. Longer-term follow-up of clinical and patient-reported outcomes will further inform the implementation of increasingly comprehensive genetic testing in clinical practice.

## Data Availability

The following represents disclosure information provided by authors of this manuscript. All relationships are considered compensated. Relationships are self-held unless noted. I = Immediate Family Member, Inst = My Institution. Relationships may not relate to the subject matter of this manuscript. For more information about ASCO's conflict of interest policy, please refer to www.asco.org/rwc or ascopubs.org/po/author-center. **Research Funding:** Myriad Genetics (Inst) **Research Funding:** Myriad Genetics (Inst) **Other Relationship:** Ambry Genetics, Color Genomics, GeneDx, BioReference, InVitae, Genentech **Research Funding:** Myriad Genetics (Inst) **Consulting or Advisory Role:** PWNHealth **Research Funding:** Myriad Genetics (Inst) **Research Funding:** Myriad Genetics (Inst), Natera (Inst) **Employment:** Myriad Genetics, GRAIL **Stock and Other Ownership Interests:** Myriad Genetics, GRAIL **Employment:** Oxford Immunotec, Myriad Genetics **Leadership:** Oxford Immunotec, Myriad Genetics **Stock and Other Ownership Interests:** Oxford Immunotec, Myriad Genetics **Patents, Royalties, Other Intellectual Property:** Founding patent holder, AssurexHealth, since 2016, a wholly owned subsidiary of Myriad Genetics **Employment:** Myriad Genetics **Leadership:** Myriad Genetics **Stock and Other Ownership Interests:** Myriad Genetics **Consulting or Advisory Role:** Protean Biodiagnostics **Travel, Accommodations, Expenses:** Myriad Genetics **Employment:** Myriad Genetics **Employment:** Myriad Genetics **Consulting or Advisory Role:** Myriad Genetics **Employment:** Myriad Genetics **Stock and Other Ownership Interests:** Myriad Genetics **Travel, Accommodations, Expenses:** Myriad Genetics **Consulting or Advisory Role:** Brogent International **Research Funding:** Myriad Genetics **Stock and Other Ownership Interests:** Universal Dx, Lean Medical **Consulting or Advisory Role:** Motus GI, Medtronic/Covidien (Given), Quorum Consulting, Clinical Genomics **Research Funding:** Genentech (Inst), AstraZeneca (Inst), Puma Biotechnology (Inst), Myriad Genetics (Inst) **Employment:** Brogent International **Leadership:** Brogent International **Stock and Other Ownership Interests:** Brogent International, Fulgent Therapeutics **Consulting or Advisory Role:** Myriad Genetics, Fulgent Therapeutics **Research Funding:** Myriad Genetics (Inst) No other potential conflicts of interest were reported.
